# Dog characteristics and future risk of asthma in children growing up with dogs

**DOI:** 10.1038/s41598-018-35245-2

**Published:** 2018-11-15

**Authors:** Tove Fall, Sara Ekberg, Cecilia Lundholm, Fang Fang, Catarina Almqvist

**Affiliations:** 1 Department of Medical Sciences, Molecular Epidemiology and Science for Life Laboratory, Uppsala University, Uppsala, Sweden; 2 Department of Medical Epidemiology and Biostatistics, Karolinska Institutet, Stockholm, Sweden; 3 Unit of Pediatric Allergy and Pulmonology at Astrid Lindgren Children’s Hospital, Karolinska University Hospital, Stockholm, Sweden

## Abstract

There is observational evidence that children exposed to dogs in early life are at lower risk of asthma. It is unknown whether this association is modified by dog characteristics such as sex, breed, number of dogs, and dog size. The aim of this study was to determine whether different dog characteristics modify the risk of asthma among children exposed to dogs during their first year of life. In the main analysis, we used national register data for all children born in Sweden from Jan 1st 2001 to Dec 31st 2004 with a registered dog in the household during their first year of life (n = 23,585). We used logistic regression models to study the association between dog characteristics and the risk of asthma or allergy diagnosis and medication at age six. The prevalence of asthma at age six was 5.4%. Children exposed to female dogs had lower risk of asthma compared to those exposed to male dogs, odds ratio, OR = 0.84 (95% confidence interval, CI 0.74 to 0.95). Children with two dogs or more had lower risk of asthma than those with one dog only, OR = 0.79 (95% CI 0.65 to 0.95). Children whose parents had asthma and allergy had a higher frequency of exposure to dog breeds anecdotally described as “hypoallergenic” compared to those parents without asthma or allergy (11.7% vs 7.6%, p < 0.001). Exposure to these breeds were associated with higher risk of allergy OR = 1.27 (95% CI 1.02 to 1.59) but not asthma. In conclusion, we found evidence of an association between the sex of dog and the number of dogs with a lower risk of childhood asthma in dog-exposed children.

## Introduction

Childhood asthma is a global public health concern. About 54% of affected patients are sensitized to mammalian allergens at age 19, compared to about 21% of non-asthmatic individuals^1^ and many affected children suffer from asthma exacerbation after pet exposure^2^. However, increasing evidence shows that exposure to dogs during early childhood is associated with lower risk of asthma, for example, a 13% risk reduction in our recent study including 276,298 children^3^. The reason for this inverse relationship is not clear, and several factors may come into play. Children in dog households are more exposed to microbial materials such as endotoxins^4^, which could modulate the immune system and respiratory epithelium^5^. They may also have a beneficial life-style such as spending more time outdoors^6^. There is also a risk of avoidance bias, where families with high predisposition to allergy do not acquire a furred pet to the same extent as those without^7^, but are meanwhile exposed to pet allergen indirectly which may induce sensitization and symptoms^8,9^. The role of early exposure to antigens in the home environment for tolerance development is not clear.

However, life-style or dog management varies also among dog-owning families. House dust levels of a major dog allergen, Can f 1, has been shown to vary with the time the dog is kept indoors^10^. Sex of the dog and breed may also affect shedding of allergens and endotoxins. A recently reported major dog allergen, Can f 5, is excreted from prostate tissue into urine of male dogs^11^, and its expression is reduced in neutered males^12^. In Sweden, only about 4–7% of dogs were neutered in 1999^13^, a rate that had increased to 22% in 2012^14^. Furthermore, some dog breeds with a non-shedding coat are anecdotally described as being “hypoallergenic” and some of these are also enlisted by the American Kennel Club Association (AKC) as suitable for people with allergies^15^. There is however little scientific evidence that such breeds have lower allergen levels and two studies failed to show any difference among dog breed groups in allergen shedding^16,17^. Further, neither number of dogs nor the weight of the dog was associated with the amount of Can f 1 allergen in the household^10^, but these factors may affect other potentially protecting parameters. A previous prospective birth cohort showed that children exposed to two or more dogs or cats were less likely to have allergic sensitization at 6 to 7 years of age, while having only one pet was not^18^.

The impact of dog characteristics on future asthma in children exposed to dogs in early life is not previously reported. Here, we use national registers of dog ownership combined with child and parental health data to assess whether dog characteristics such as sex, breed, number of dogs or size of the dog modifies the future risk of asthma among children exposed to dogs during their first year of life.

## Methods

### Study population and study design

We identified two cohorts of Swedish children by linking data from Register of the Total Population to the Medical Birth Register via individual personal identification numbers. The cohort used in the main analysis was born from January 1 2001 to December 31 2004 and was assessed for any asthma event at age six (during their 7th year of life). A second younger cohort used for sensitivity analysis was born from July 1 2005 to December 31 2010 and was followed from age 1 until first asthma diagnosis, death, emigration or December 31 2011, whichever occurred first.

Dog ownership was defined by linking parental personal identity numbers with the Swedish Board of Agriculture National Dog Register and the Swedish Kennel Club’s Register. In a survey by Statistics Sweden from 2012 the registration rate was estimated as 83% (95% confidence interval, 78–87%)^14^. Only children having a parent registered as a dog owner during the child’s whole first year of life were included in the main analysis of the present study as the comparison with non-owners was already covered in a previous study^3^.

For follow-up and ascertainment of asthma outcomes, we crossed linked both cohorts to the Cause of Death Register, the Migration Register, the National Swedish Patient Register (NPR) and the Swedish Prescribed Drug Register (SPDR).

The regional ethical board in Stockholm, Sweden, approved this study and allowed the researchers to waive the requirement for obtaining informed consent or parental permission.

### Exposure

Dog exposure characteristics were assessed during children’s first year of life and included sex of dog, breed, number of dogs and size of dog. The information about dogs’ dates of death was incomplete and we therefore used a maximum age of 10 years for dogs with missing death date. If the same dog appeared in both registers we used the earliest registration date as date of owner registration and in case of contradicting information we used the Swedish Kennel Club’s data. The registers do not contain information on castration status of the dog so only male/female categories were assessed. Breeds were categorized according to the Fédération Cynologique Internationale (FCI). We also classified breeds as “hypoallergenic - web definition” from Nicholas *et al*.^17^, who used the key words “hypoallergenic dogs” or “hypoallergenic dog breeds” in a systematic web search. As an alternative categorization breeds identified as hypoallergenic by the AKC^15^ were defined as “hypoallergenic – AKC definition”. Dog size was based on average breed size specified by the Swedish Kennel Club and categorized into small (<40 cm), medium (40–60 cm) and large (>60 cm) size. Mixed breed dogs were not included in those analyses.

### Outcome definition

In brief, asthma and allergy was defined at age six and up to age five by criteria validated by Örtqvist *et al*.^19^ and Henriksen *et al*.^20^, based on data from NPR and the SPDR (see Supplementary information for details).

### Potential confounders and effect modifiers

We considered maternal age, parental birth country, parental education, parental asthma, parental allergy, and population density as potential confounders, whereas parental asthma and allergies were considered as potential effect modifiers. We also adjusted all models for sex of dog and number of dogs in the household, as dog breeders usually keep many female dogs, which could bias estimates of the other characteristics. Parental educational level based on data from the Longitudinal Integration Database for Health Insurance and Labour Market Studies (LISA) as the highest educated parent (middle school, high school, college (<3 years), college graduates or higher). Parental asthma and allergy was denoted if the biological mother or father fulfilled the criteria for asthma/allergy defined in the Supplementary information at any time during the study period. Population density was calculated as the number of habitants/km^2^ in the child’s birth parish in 2001.

### Statistical analysis

Stata 14.1 was used for all statistical analyses. Standard errors were adjusted for dependent observations (siblings) using a sandwich estimator.

#### Main analysis

We estimated the association between dog exposure characteristics and prevalent asthma and allergy at age six using a series of multiple logistic regression models with asthma/allergy as the dependent variable and each dog characteristic variable (sex, breed, ‘hypoallergenic breed’, number of dogs, size of dog) as an independent variable adjusting for potential confounders. We further assessed the association of dog ownership with “allergic asthma”, “non-allergic asthma” and allergies as three separate outcomes. In secondary analysis, we stratified the analysis based on parental asthma and allergy. The χ^2^ test was used to assess differences in dog characteristics between families with and without parental asthma and allergies.

#### Sensitivity analyses

For the younger cohort we used Cox proportional hazards model with age as the underlying time scale, adjusting for the same confounders. The assumption about proportional hazards was formally tested using Therneau and Grambsch test of the Schoenfeld residuals.

We also analyzed the dataset restricted to only first-borns as the choice of ownership in this group cannot be affected by sibling’s diseases.

#### Comparison to non-exposed children

In our previous study of dog ownership and risk of childhood asthma^3^, we used slightly different criteria for asthma, and we therefore calculated the prevalence of asthma at age six in non-dog exposed children born 2001–2004 and the incidence of asthma in non-dog exposed children born 2005–2010, to provide information on the background risk using the updated criteria. We also calculated the odds ratio of asthma in relation to some dog characteristics using *non-dog exposed* children as the reference groups in logistic regression models adjusted for the same confounders.

## Results

The analytical dataset (Table 1) included 23,585 dog-exposed children born during 2001–2004 for the main analysis and 62,333 dog-exposed children born during July 1 2005–2010 for the sensitivity analyses. The register of dogs from the National Board of Agriculture had increasing number of newly registered dogs during the study period, while the Swedish Kennel Club had comparable number of new registrations each year. Since cross-breeds are most often registered only in the National Board of Agriculture, the proportion of cross-breeds is higher in later years. In the main cohort, 8.4% had a breed fulfilling the “hypoallergenic – web definition” and 4.7% according to the “hypoallergenic – AKC definition”. Seventeen percent of children in the main cohort had at least one parent who fulfilled the criterion for parental asthma and 31% for parental allergy during the study period (Table 1).

**Table 1:** Descriptive information of the dataset for children born 2001–2004 (n = 23,585) and for the dataset of children born July 2005–2010 (n = 62,333).

		Old cohort	Young Cohort^a^
		n (%)	n (%)
Breed group	Sheepdogs and Cattledogs	3317 (14.1)	5 039 (8.1)
	Pinscher and Schnauzer - Molossoid	3801 (16.1)	8 455 (13.6)
	Terriers	1421 (6.0)	5 184 (8.3)
	Dachshunds	672 (2.8)	911 (1.5)
	Spitz and primitive types	1778 (7.5)	3 504 (5.6)
	Scent hounds and related breeds	1645 (7.0)	2 522 (4.0)
	Pointing Dogs	999 (4.2)	1 634 (2.6)
	Retrievers - Flushing Dogs - Water Dogs	4788 (20.3)	9 377 (15.0)
	Companion and Toy Dogs	1433 (6.1)	4 711 (7.6)
	Sighthounds	266 (1.1)	688 (1.1)
	Cross-breed	1360 (5.8)	12 246 (19.6)
	>1 group	2105 (8.9)	8 062 (12.9)
Hypoallergenic – web definition^b^	Yes	1877 (8.4)	5 370 (10.7)
	Both	589 (2.7)	4 595 (9.2)
Hypoallergenic - AKC definition^b^	Yes	1038 (4.7)	2 663 (5.3)
	Both	454 (2.0)	4 125 (8.2)
Number of dogs	≥2 dogs	5355 (22.7)	16245 (26.1)
Dog Size^b^	40 cm	4 819 (21.7)	13 513 (27.1)
	40–60 cm	11 075 (49.8)	20 392 (41.0)
	>60 cm	4 587 (20.6)	8 664 (17.4)
	>1 size	1 744 (7.8)	7 215 (14.5)
Dog Sex	male	12 123 (51.4)	31 192 (50.0)
	female	9076 (38.5)	23 746 (38.1)
	both	2386 (10.1)	7 383 (11.8)
	missing	0 (0.0)	12 (0.0)
Maternal age	<25	3267 (13.8)	11 407 (18.3)
	25–29	8530 (36.2)	20 738 (33.3)
	30–34	7734 (32.8)	18 989 (30.5)
	≥35	4054 (17.2)	11 206 (18.0)
Parents highest education	Middle school	606 (2.6)	2 252 (3.6)
	High school	12 909 (54.7)	31 833 (51.1)
	College (<3 years)	3963 (16.8)	8 550 (13.7)
	College graduates or higher	6101 (25.9)	19 661 (31.5)
	Missing	6 (0.0)	37 (0.1)
Parents birth country	Nordic	22 157 (93.9)	56 511 (90.6)
	Non-nordic	1272 (5.4)	5 175 (8.3)
	Missing	156 (0.7)	647 (1.0)
Parental asthma	Yes	4014 (17.0)	10 425 (16.7)
Parental allergy	Yes	7257 (30.8)	19 966 (32.0)

^a^Excluding those leaving study before age 1. ^b^Only pure-breed dogs included in analysis.

AKC – American Kennel Club.

In fully adjusted models, children exposed to female dogs during their first year of life were found to be at lower risk for asthma at age six than those exposed to male dogs, adjusted OR 0.84 (0.74, 0.95) (Table 2). Children with two dogs or more in their homes had lower risk of asthma than those with one dog only, adjusted OR 0.79 (0.65, 0.95). No association was found for the size of dog with asthma although there was a trend for lower OR with increasing size of dog.

**Table 2:** Logistic regression models assessing the association of dog characteristics in 23,425 dog-exposed children and asthma diagnosis at age six with complete information on confounders.

	Asthma n (%)	OR (95% CI)	OR^a^ (95% CI)
**Dog sex**			
male	701 (5.8)	1	1
female	442 (4.9)	**0**.**84 (0**.**74**, **0**.**95)**	**0**.**84 (0**.**74**, **0**.**95)**
both	129 (5.4)	0.93 (0.76, 1.13)	1.10 (0.83, 1.46)
**Breed group**			
Sheepdogs and Cattledogs	157 (4.8)	0.85 (0.69, 1.04)	**0**.**80 (0**.**65**, **0**.**99)**
Pinscher and Schnauzer - Molossoid	227 (6.0)	1.09 (0.90, 1.31)	1.04 (0.86, 1.26)
Terriers	78 (5.5)	0.99 (0.76, 1.29)	0.95 (0.73, 1.25)
Dachshunds	31 (4.6)	0.82 (0.56, 1.21)	0.82 (0.56, 1.20)
Spitz and primitive types	103 (5.8)	1.05 (0.82, 1.33)	1.04 (0.81, 1.33)
Scent hounds and related breeds	76 (4.6)	0.82 (0.63, 1.07)	0.82 (0.63, 1.07)
Pointing Dogs	45 (4.5)	0.80 (0.58, 1.12)	0.80 (0.58, 1.12)
Retrievers - Flushing Dogs - Water Dogs	265 (5.6)	1	1
Companion and Toy Dogs	113 (8.0)	**1**.**47 (1**.**16**, **1**.**85)**	**1**.**29 (1**.**02**, **1**.**64)**
Sighthounds	12 (4.6)	0.81 (0.45, 1.46)	0.77 (0.42, 1.42)
Cross-breed	75 (5.6)	1.00 (0.77, 1.30)	0.96 (0.73, 1.25)
More than one group	90 (4.3)	**0**.**76 (0**.**59**, **0**.**97)**	**0**.**72 (0**.**53**, **0**.**97)**
**Hypoallergenic– web definition**			
No	1 044 (5.3)	1	1
Yes	124 (6.6)	**1**.**27 (1**.**04**, **1**.**54)**	1.20 (0.98, 1.47)
Both	29 (5.0)	0.93 (0.63, 1.37)	0.96 (0.64, 1.44)
**Hypoallergenic – AKC definition**			
No	1 105 (5.4)	1	1
Yes	70 (6.8)	**1**.**28 (1**.**00**, **1**.**65)**	1.17 (0.91, 1.51)
Both	22 (4.9)	0.90 (0.58, 1.42)	0.93 (0.58, 1.49)
**Number of dogs**			
1	1012 (5.6)	1	1
>1	260 (4.9)	**0**.**87 (0**.**75**, **1**.**00)**	**0**.**79 (0**.**65**, **0**.**95)**
**Dog size**			
Small (<40 cm)	292 (6.1)	1.14 (0.99, 1.32)	1.10 (0.95, 1.28)
Medium (40–60 cm)	592 (5.4)	1	1
Large (>60 cm)	237 (5.2)	0.97 (0.83, 1.13)	0.95 (0.81, 1.11)
>one size	76 (4.4)	0.80 (0.62, 1.04)	0.82 (0.62, 1.09)

^a^Adjusted for maternal age (<25, 25–29, 30–34, ≥35), Parents’ birth country (Nordic/non-Nordic), parental education (max) (Middle school, High school, College (<3 yrs), College graduates or higher), parental asthma (yes/no), parental allergy (yes/no), population density (per km^2^), dog sex and number of dogs in the family.

In unadjusted models, children exposed to breeds labelled as “hypoallergenic – web definition” had higher risk of asthma at age six compared to children exposed to other breeds, OR 1.27 (1.04, 1.54), but this association was attenuated in the adjusted model with an OR of 1.20 (0.98, 1.47). Children exposed to dogs classified as “Sheepdogs and Cattledogs” were at decreased risk of asthma at age six, adjusted OR 0.80 (0.65, 0.99) and those exposed to “companion and toy dogs” were at increased risk of asthma, adjusted OR 1.29 (1.02, 1.64), compared to the largest breed group “Retrievers - Flushing Dogs - Water Dogs”.

In the analysis of “allergic asthma”, “non-allergic asthma” and allergies as three separate outcomes (eTable 1), having a “hypoallergenic – web definition” dog was associated with increased risk of allergy, OR 1.27 (1.02, 1.59).

We further stratified the analysis by parental asthma and allergy. The proportion of families with male or female dogs did not differ by parental asthma or allergy (eTable 2, p = 0.82). We found that the inverse association of female dog exposure with childhood asthma was most prominent in the group of children with at least one parent that fulfilled both the asthma and the allergy criteria, adjusted OR 0.68 (0.50 to 0.91) (Table 3). Parental asthma and allergies was associated with having a “hypoallergenic” breed with both definitions, during the first year of life (eTable 2, p < 0.001). Pair-wise testing revealed that children in families in which one or both parents had asthma and allergy or allergy only had a higher frequency (11.7% and 9.8%) of dog breeds described as “hypoallergenic – web definition” compared to families with no parental asthma or allergy (7.6%, p = <0.001). Similar patterns were noted for “hypoallergenic – AKC definition”.

**Table 3:** Stratified logistic regression models assessing the association of dog characteristics in dog-exposed children and asthma diagnosis at age six.

	No Parental Asthma or Allergy		Parental Allergy (no asthma)		Parental Asthma (no allergy)		Parental Asthma AND Allergy	
	Asthma n (%)	OR^a^ (95% CI)	Asthma n (%)	OR^a^ (95% CI)	Asthma n (%)	OR^a^ (95% CI)	Asthma n (%)	OR^a^ (95% CI)
**Dog sex**								
male	300 (4.0)	1	167 (6.5)	1	83 (9.6)	1	151 (12.9)	1
female	205 (3.7)	0.91 (0.76, 1.09)	113 (6.0)	0.91 (0.71, 1.17)	45 (7.3)	0.75 (0.52, 1.10)	79 (9.0)	**0.68 (0.50, 0.91)**
both	56 (3.9)	1.17 (0.82, 1.67)	30 (6.3)	1.15 (0.74, 1.79)	15 (6.8)	0.85 (0.46, 1.55)	28 (11.6)	1.09 (0.66, 1.80)
**Breed group**								
Sheepdogs and Cattledogs	71 (3.5)	0.88 (0.65, 1.20)	36 (5.2)	0.72 (0.48, 1.09)	21 (8.3)	0.90 (0.51, 1.61)	29 (8.9)	0.67 (0.41, 1.10)
Pinscher and Schnauzer - Molossoid	104 (4.4)	1.13 (0.86, 1.49)	59 (7.4)	1.06 (0.73, 1.52)	22 (8.7)	0.94 (0.52, 1.67)	42 (11.4)	0.88 (0.56, 1.39)
Terriers	38 (4.5)	1.17 (0.80, 1.72)	18 (5.4)	0.78 (0.45, 1.34)	5 (5.1)	0.55 (0.21, 1.47)	17 (12.0)	0.95 (0.52, 1.72)
Dachshunds	9 (2.1)	0.54 (0.28, 1.07)	6 (4.3)	0.60 (0.26, 1.37)	9 (16.4)	2.06 (0.87, 4.84)	7 (12.5)	0.95 (0.42, 2.15)
Spitz and primitive types	59 (5.2)	1.36 (0.97, 1.89)	13 (3.9)	0.54 (0.29, 1.03)	16 (10.0)	1.13 (0.60, 2.15)	15 (10.6)	0.81 (0.43, 1.51)
Scent hounds and related breeds	30 (2.8)	0.71 (0.47, 1.06)	26 (7.7)	1.12 (0.70, 1.80)	7 (7.2)	0.79 (0.34, 1.84)	13 (9.6)	0.72 (0.36, 1.44)
Pointing Dogs	23 (3.5)	0.93 (0.58, 1.49)	11 (5.6)	0.81 (0.42, 1.54)	4 (7.8)	0.87 (0.29, 2.55)	7 (7.2)	0.53 (0.23, 1.21)
Retrievers - Flushing Dogs - Water Dogs	116 (3.8)	1	69 (6.9)	1	28 (8.8)	1	52 (12.7)	1
Companion and Toy Dogs	39 (5.2)	1.34 (0.93, 1.95)	28 (8.5)	1.24 (0.77, 2.01)	12 (11.2)	1.29 (0.64, 2.61)	34 (14.8)	1.22 (0.74, 2.02)
Sighthounds	6 (3.8)	0.97 (0.42, 2.25)	5 (9.4)	1.39 (0.54, 3.60)	1 (4.8)	0.54 (0.07, 4.32)	0 (0.0)	1.00 (1.00, 1.00)
Cross-breed	31 (3.9)	0.98 (0.66, 1.48)	18 (6.0)	0.85 (0.50, 1.46)	9 (8.0)	0.87 (0.40, 1.89)	17 (12.6)	0.99 (0.55, 1.78)
More than one group	35 (2.7)	0.69 (0.45, 1.05)	21 (5.1)	0.72 (0.43, 1.23)	9 (5.2)	0.56 (0.26, 1.24)	25 (11.4)	0.89 (0.51, 1.57)
**Hypoallergenic– web definition**								
No	467 (3.8)	1	254 (6.2)	1	121 (8.6)	1	202 (11.1)	1
Yes	53 (5.1)	**1.37 (1.02, 1.84)**	32 (7.0)	1.16 (0.78, 1.73)	9 (7.6)	0.89 (0.44, 1.79)	30 (11.9)	1.11 (0.74, 1.67)
Both	10 (2.9)	0.85 (0.42, 1.70)	6 (6.0)	1.04 (0.44, 2.46)	4 (5.9)	0.76 (0.27, 2.10)	9 (11.8)	1.26 (0.61, 2.61)
**Hypoallergenic – AKC definition**								
No	493 (3.8)	1	271 (6.3)	1	126 (8.6)	1	215 (11.1)	1
Yes	27 (4.9)	1.31 (0.88, 1.95)	17 (6.9)	1.10 (0.65, 1.87)	6 (8.2)	0.96 (0.41, 2.24)	20 (12.3)	1.14 (0.70, 1.85)
Both	10 (3.8)	1.09 (0.54, 2.19)	4 (4.9)	0.83 (0.30, 2.33)	2 (4.4)	0.56 (0.14, 2.34)	6 (10.0)	1.03 (0.43, 2.47)
**Number of dogs**								
1	449 (4.0)	1	252 (6.5)	1	114 (9.0)	1	197 (11.2)	1
>1	112 (3.4)	0.83 (0.67, 1.03)	58 (5.6)	0.84 (0.62, 1.13)	29 (6.7)	0.72 (0.47, 1.09)	61 (11.5)	1.04 (0.75, 1.43)
**Dog size**								
Small (<40 cm)	119 (4.1)	1.05 (0.84, 1.31)	75 (7.2)	1.21 (0.90, 1.63)	33 (10.2)	1.15 (0.74, 1.78)	65 (12.0)	1.07 (0.77, 1.50)
Medium (40–60 cm)	273 (3.9)	1	138 (6.0)	1	71 (8.8)	1	110 (11.2)	1
Large (>60 cm)	106 (3.7)	0.93 (0.74, 1.18)	65 (6.8)	1.15 (0.84, 1.56)	23 (7.4)	0.79 (0.49, 1.29)	43 (9.6)	0.83 (0.57, 1.21)
>one size	32 (3.0)	0.79 (0.53, 1.19)	14 (4.3)	0.75 (0.42, 1.34)	7 (4.7)	0.53 (0.24, 1.17)	23 (12.2)	1.20 (0.71, 2.03)

^a^Adjusted for maternal age (<25, 25–29, 30–34, ≥35), parents’ birth country (Nordic/non-Nordic), parental education (max) (Middle school, High school, College (<3 yrs), College graduates or higher), population density (per km^2^).

### Sensitivity analyses

Analysis in the younger cohort of pre-school children did not show any evidence of associations of dog characteristics with asthma (eTable 3).

In the older cohort, analyses restricted to the 10,954 first-born children showed attenuated or abolished associations for female dogs, OR 0.95 (0.80 to 1.13) and >1 dog 0.88 (0.68, 1.15). The associations for breed were similar (eTable 4).

### Comparison to non-exposed children

The overall prevalence of asthma at age six was 5.95% in non-exposed children, and 5.43% in dog-exposed children. Having a male dog compared to no dog yielded an OR of 0.94 (0.87, 1.02). Having a dog classified as “hypoallergenic – web definition” compared to no dog yielded an OR of 1.01 (0.84, 1.21), and “hypoallergenic – AKC definition” an OR of 1.00 (0.79, 1.26).

## Discussion

Our main finding was that among dog-exposed children, children with female dogs had less asthma at age six than children with only male dogs. Further, we found that children with two or more dogs in the home had less asthma than those with one dog only. Having a breed regarded as “hypoallergenic” was more common in families with parental allergy and was associated with allergy, but not asthma, in children at age six. These findings are novel and have not been previously reported.

Childhood asthma is a heterogeneous disorder. In school age, the condition is associated with sensitization to airborne allergens in the majority of cases, such as to allergens from furred animals. It is debated if early exposure to antigens affects the risk later respiratory disease^21,22^. In our study, we found that children exposed to female dogs had about 16% lower risk of asthma. This is unlikely to be a result of a selection bias, supported by the fact that similar proportion of female dogs were reported among households with and without parental asthma and allergy phenotypes. We speculate that the differences in allergen excretion from intact male dogs to other dogs may come into play. The recently reported major dog allergen, Can f 5 (analogous to the human PSA), is excreted from prostate tissue into urine of male dogs^11^. In Sweden, relatively few dogs are castrated and it is not possible to generalize our results to children exposed to male dogs in countries where castration rates differ. To avoid overlap with our previous study in this data^3^, we focused on the group of children that all had dogs. However, we compared the risk of asthma in children exposed to male dogs to that of children not exposed to any dogs and found similar risks of asthma (OR 0.94, 0.87, 1.02), indicating that ownership of a male dog is not associated with increased risk of asthma compared to children without dogs.

We found that children exposed to two dogs or more in the first year of life had approximately 21% lower risk of asthma than those with only one dog. In a previous study, we showed that dog exposure is associated with a 13% lower risk of asthma in the general child population^3^, which could be explained by differences in exposure to microbial material, or differences in lifestyle such as a more active outdoor lifestyle. These factors could be pronounced in a multiple-dog household. However, having two dogs or more could also be indicative of a family without any previous problems with allergic or asthmatic disease. We did not see any association of size of the dog with risk of asthma, although the point estimates were lower for each increasing size.

To assess whether the breed affects the risk of asthma in the child, we performed two sets of analyses. The first compared different groups of breeds to the most common breed group, “Retrievers - Flushing Dogs - Water Dogs”. We found that the risk of prevalent asthma was about 29% higher in children exposed to “Companion and Toy Dogs” and about 20% lower in those exposed to “Sheepdogs and Cattledogs”. Although we adjusted for population density in the area of residence, there might be residual confounding explaining this association. Farmers often keep “Sheepdogs and Cattledogs”, and farm children are known to have a strongly reduced risk of asthma. Further, “Companion and Toy Dogs” are common in cities, where asthma is also more prevalent. It is also likely that farm dogs in general spend more time outdoors than a small companion dog, which may influence the amount of endotoxins and other microbial products in the household, further influencing influence the risk of asthma^5^.

We categorized dogs with two previously proposed definitions for “hypoallergenic” breeds^17^, one based on a web search, encompassing 45 breeds, and the other one based on 23 breeds enlisted by the AKC. We found that these types of breeds were more common in families where at least one parent fulfilled the criteria for allergy, with or without asthma, indicating that some families take into account these recommendations when choosing a dog. We did not see a clear association of exposure to “hypoallergenic” dogs with risk of asthma, but with an increased risk of allergy. Previous studies have not shown differences among dog breed groups in allergen shedding^16,17^, and we speculate that the association is confounded by parental allergy to furry animals, which increases the risk of allergy in their children and thus the likelihood of acquiring a “hypoallergenic” dog^7^. We were only able to adjust for parental allergy medication, but not the type of allergy, which could explain the remaining association after accounting for parental allergy.

The results from the younger cohort did not reveal association of any of the dog characteristics with incident asthma in preschool age. This discrepancy could be due to differences in the asthma phenotype, where wheezing in preschool children is more often transient or related to viral infections. It could also be explained be differences in dog registration patterns and the level of castration over time. Moreover, some of the identified associations was attenuated in the subgroup analysis of first-borns, although with overlapping confidence intervals to the main analysis. Whether this was caused by reduced power or by biological differences is not possible to say.

The strengths of the study include the prospective design, the nation-wide coverage of the registers and the validated outcome measurement. However, some limitations of the study should be noted. Firstly, conclusions about causal directions is difficult to do as the choice of dog breed, sex and number of dogs is not random, and can sometimes be affected by disease history in the owner’s family. Therefore, overemphasis of our results should be avoided when giving recommendations to families about dog ownership. However, as a clinical trial on these research questions is difficult or impossible to implement in the society, evidence from cohort studies such as this one may be the highest level of evidence that we will have. Secondly, children with family history of asthma and allergic disease who avoid direct dog exposure may still be exposed to ubiquitous pet allergens^9^. This may result in increased risk of symptoms, which affects many children in the population^8,9^, however in our main analysis we only included dog-exposed children. Of note is that the asthma definition was updated from our previous study^3^ and hence the prevalence was slightly higher in the present report. Lastly, we did not have information on the proportion of time the dog spent outdoor, the level of contact the dog had with the child, or whether or not cats or other furred pets were present in the household.

We conclude that within a nation-wide cohort of children exposed to dogs in their home during first year of life, several dog characteristics such as having only female dogs and increased number of dogs, but not “hypoallergenic” breeds are associated with a lower risk of asthma at age six. We also found an association of exposure to “hypoallergenic” breeds with allergy, which might be caused by a selective behaviour in families with allergies.

## Electronic supplementary material

Supplementary Information
