# Supplementary material for: Dog characteristics and future risk of asthma in children growing up with dogs
Source: Sci Rep. 2018 Nov 15;8:16899. doi: 10.1038/s41598-018-35245-2 (PMC6237975; doi:10.1038/s41598-018-35245-2)
Supplement: Supplementary file 1 — Supplementary Information [file 41598_2018_35245_MOESM1_ESM.docx]

**Supplementary Online Content**

**Dog characteristics and future risk of asthma in children growing up with dogs**

Tove Fall, PhD, Sara Ekberg, MSc, Cecilia Lundholm, MSc, Fang Fang, PhD and Catarina Almqvist, PhD,

**eMethods**

**eTable 1**. Logistic regression models assessing the association of dog characteristics in dog-exposed children and non-allergic asthma, allergic asthma and allergy at age six, respectively

**eTable 2.** Number of dogs and frequency with different characteristics in families with and without parental asthma and allergy.

**eTable 3.** Cox proportional hazards models assessing the association of dog characteristics in dog-exposed children and asthma diagnosis after age 1 in 61,653 children born July 2005-2010.

**eTable 4.** Logistic regression models assessing the association of dog characteristics in 10,954 first-born dog-exposed children and asthma diagnosis at age six.

**eMethods**

**Outcome definition**

*Asthma definition – parents and children >4.5 years*

Asthma was defined as fulfilling any of the following three criteria during 7^th^ year of life (age six) in the main analysis (older children cohort), at any time-point for parents, and for children >4.5 years in the sensitivity analysis (younger children cohort): 1) a diagnosis (10^th^ Swedish Revision of the International Classification of Diseases code: J45) from the National Patient Register; 2) ≥2 dispensed prescriptions of inhaled corticosteroids (ATC code: R03BA), fixed combinations of β2-agonists and corticosteroids (ATC code: R03AK06 or R03AK07) and/or leukotriene receptor antagonists (ATC code: R03DC03); or 3) ≥3 dispensed prescriptions of inhalations of selective β2-adrenoreceptor agonists (R03AC02, R03AC03, R03AC12, R03AC13) and any of the medications listed in 2) within a 12 months period. In the main analysis, for the second and third criterion, only one dispense of asthma medications had to be during age six.

*Children <4.5 years*

To be defined as asthmatic, children aged 0-4.5 years were required to both have an asthma diagnosis (J45) from the National Patient Register and fulfill the criteria for asthma medication from SPDR. Asthma medication in the SPDR was defined as having ≥2 dispensed prescriptions of inhaled corticosteroids (R03BA), fixed combinations of β2-agonists and corticosteroids (R03AK06 or R03AK07) and/or leukotriene receptor antagonists (R03DC03) with ≥2 weeks between prescriptions. Children were also considered as having asthma if they had ≥3 dispensed prescriptions of any of the medications listed above or inhalations of selective β2-adrenoreceptor agonists (R03AC02, R03AC03, R03AC12, R03AC13) within a 12-months period.^21^

*Allergy definition*

Allergy (allergic rhinoconjunctivitis) was assessed in the older cohort only and defined using the criteria described by Henriksen.^2^ In brief, one of the following four criteria should be fulfilled from age 5.5-7. CRITERIA 1 (based on ICD-10): ≥1 hospital contact for:  J30 “hay fever and allergic rhinitis” J30.0 ”vasomotor rhinitis” J30.1 “allergic rhinitis due to pollen” J30.2 “other seasonal allergic rhinitis” J30.3 “other allergic rhinitis” J30.4 “allergic rhinitis, unspecified” J31.0 “chronic rhinitis”. CRITERIA 2 (based on ATC and ICD-10): ≥ 2 filled prescriptions of: R01AD01 – R01AD60 “inhaled corticosteroids for rhinitis”. And no hospital contact for (exclusions criteria):   J33 “nasal polyps” J330 ”polyps in nasal cavity” J331 “polyp related sinus degeneration” J331A “woakes' ethmoiditis” J338” nasal polyps, other” J338A “polypus sinus sphenoidalis” J339 “nasal polyps, unspecified” J010- J019  ”acute sinusitis” J320 –J329  ”chronic sinusitis”. CRITERIA 3 (based on ICD-10 and ATC) ≥2 filled prescriptions of:  R06A “antihistamines for systemic use” And no hospital contact for: L29 “pruritus” or  DL50 “allergic urticaria”. CRITERIA 4 (based on ATC) ≥ 1 filled prescriptions of:  V01A “specific immune therapy, allergen substract therapy” or/and S01GX “medication for allergic conjunctivitis”.

**eTable 1. Logistic regression models assessing the association of dog characteristics in dog-exposed children and non-allergic asthma, allergic asthma and allergy at age six, respectively**

|  | **Non-allergic Asthma n (%)** | **OR (95% CI)** | **Allergic Asthma n (%)** | **OR (95% CI)** | **Allergy n(%)** | **OR (95% CI)** |
| --- | --- | --- | --- | --- | --- | --- |
| **Dog sex** |  |  |  |  |  |  |
| male | 535 (4.4) | 1.00 | 166 (1.4) | 1.00 | 499 (4.1) | 1.00 |
| female | 345 (3.8) | **0.86 (0.75,0.99**) | 97 (1.1) | 0.79 (0.61,1.02) | 320 (3.6) | **0.86 (0.74,0.99)** |
| both | 106 (4.5) | 1.15 (0.84,1.58) | 23 (1.0) | 0.94 (0.53,1.67) | 73 (3.1) | 0.87 (0.63,1.20) |
| **Breed group** |  |  |  |  |  |  |
| Sheepdogs and Cattledogs | 123 (3.7) | 0.81 (0.64,1.02) | 34 (1.0) | 0.78 (0.51,1.19) | 105 (3.2) | 0.80 (0.63,1.03) |
| Pinscher and Schnauzer - Molossoid | 173 (4.6) | 1.03 (0.83,1.27) | 54 (1.4) | 1.10 (0.75,1.60) | 168 (4.5) | 1.13 (0.91,1.41) |
| Terriers | 59 (4.2) | 0.93 (0.69,1.26) | 19 (1.3) | 1.04 (0.62,1.76) | 56 (4.0) | 0.97 (0.72,1.32) |
| Dachshunds | 24 (3.6) | 0.81 (0.53,1.24) | 7 (1.0) | 0.82 (0.38,1.78) | 21 (3.1) | 0.81 (0.51,1.27) |
| Spitz and primitive types | 78 (4.4) | 1.01 (0.76,1.33) | 25 (1.4) | 1.14 (0.70,1.87) | 66 (3.7) | 0.99 (0.74,1.34) |
| Scent hounds and related breeds | 57 (3.5) | 0.79 (0.58,1.07) | 19 (1.2) | 0.93 (0.55,1.57) | 58 (3.5) | 0.92 (0.68,1.24) |
| Pointing Dogs | 34 (3.4) | 0.78 (0.53,1.14) | 11 (1.1) | 0.90 (0.47,1.73) | 33 (3.3) | 0.85 (0.58,1.26) |
| Retrievers - Flushing Dogs - Water Dogs | 206 (4.3) | 1 | 59 (1.2) | 1 | 186 (3.9) | 1 |
| Companion and Toy Dogs | 84 (5.9) | 1.26 (0.96,1.65) | 29 (2.0) | 1.41 (0.90,2.21) | 84 (5.9) | **1.39 (1.06,1.82)** |
| Sighthounds | 10 (3.8) | 0.82 (0.42,1.59) | 2 (0.8) | 0.61 (0.15,2.54) | 13 (4.9) | 1.27 (0.71,2.27) |
| Cross-breed | 62 (4.6) | 1.04 (0.77,1.39) | 13 (1.0) | 0.70 (0.38,1.29) | 46 (3.4) | 0.85 (0.61,1.18) |
| More than one group | 76 (3.6) | 0.77 (0.56,1.07) | 14 (0.7) | 0.53 (0.27,1.04) | 56 (2.7) | 0.72 (0.51,1.03) |
| **Hypoallergenic - web definition** |  |  |  |  |  |  |
| No | 800 (4.1) | 1.00 | 244 (1.2) | 1.00 | 734 (3.7) | 1.00 |
| Yes | 100 (5.4) | **1.27 (1.02,1.59)** | 24 (1.3) | 0.97 (0.63,1.48) | 95 (5.1) | **1.27 (1.02,1.59)** |
| Both | 24 (4.1) | 1.02 (0.65,1.61) | 5 (0.9) | 0.73 (0.29,1.84) | 17 (2.9) | 0.84 (0.51,1.38) |
| **Hypoallergenic – AKC definition** |  |  |  |  |  |  |
| No | 850 (4.1) | 1.00   \| 1.27 (1.02,1.59) \| \| --- \| \| 1.02 (0.65,1.61) \| | 255 (1.2) | 1.00 | 774 (3.8) | 1.00 |
| Yes | 55 (5.3) | 1.21 (0.91,1.61) | 15 (1.5) | 1.04 (0.62,1.76) | 58 (5.6) | **1.37 (1.03,1.80)** |
| Both | 19 (4.2) | 1.05 (0.63,1.73) | 3 (0.7) | 0.56 (0.17,1.79) | 14 (3.1) | 0.90 (0.52,1.55) |
| **Number of dogs** |  |  |  |  |  |  |
| 1 | 779 (4.3) | 1.00 | 233 (1.3) | 1.00 | 717 (4.0) | 1.00 |
| >1 | 207 (3.9) | **0.79 (0.64,0.97)** | 53 (1.0) | 0.80 (0.54,1.17) | 175 (3.3) | 0.90 (0.72,1.11) |
| **Dog size** |  |  |  |  |  |  |
| Small (<40 cm) | 222 (4.6) | 1.11 (0.94,1.31) | 70 (1.5) | 1.08 (0.80,1.44) | 199 (4.2) | 1.03 (0.87,1.23) |
| Medium (40-60 cm) | 451 (4.1) | 1 | 141 (1.3) | 1 | 425 (3.9) | 1 |
| Large (>60 cm) | 189 (4.2) | 0.99 (0.83,1.18) | 48 (1.1) | 0.79 (0.57,1.11) | 170 (3.7) | 0.95 (0.79,1.14) |
| >one size | 62 (3.6) | 0.86 (0.62,1.19) | 14 (0.8) | 0.68 (0.37,1.28) | 52 (3.0) | 0.87 (0.62,1.22) |

^a^Adjusted for maternal age (<25, 25-29,30-34,≥35), parents' birth country (Nordic/non-nordic), parental education (max) (Middle school, High school, College(<3yrs), College graduates or higher), parental asthma (yes/no), parental allergy (yes/no), population density (per km^2^), dog sex and number of dogs in the family when applicable

**eTable 2. Number of dogs and frequency with different characteristics in families with and without parental asthma and allergy.**

|  | **No Parental Asthma or Allergy** | **Parental Allergy  (no asthma)** | **Parental Asthma  (no allergy)** | **Parental Asthma AND Allergy** |
| --- | --- | --- | --- | --- |
| **Dog sex** |  |  |  |  |
| male | 7453 (51.4) | 2555 (51.8) | 862 (50.7) | 1174 (51.2) |
| female | 5616 (38.7) | 1896 (38.5) | 618 (36.4) | 875 (38.2) |
| both | 1435 (9.9) | 479 (9.7) | 220 (12.9) | 242 (10.6) |
| **Hypoallergenic -web definition** |  |  |  |  |
| No | 12320 (89.9) | 4077 (88.0) | 1401 (88.3) | 1828 (84.8) |
| Yes | 1043 (7.6) | 454 (9.8) | 118 (7.4) | 252 (11.7) |
| Both | 341 (2.5) | 100 (2.2) | 68 (4.3) | 76 (3.5) |
| **Hypoallergenic – AKC definition** |  |  |  |  |
| No | 12891 (94.1) | 4301 (92.9) | 1469 (92.6) | 1933 (89.7) |
| Yes | 549 (4.0) | 248 (5.4) | 73 (4.6) | 163 (7.6) |
| Both | 264 (1.9) | 82 (1.8) | 45 (2.8) | 60 (2.8) |

**eTable 3. Cox proportional hazards models assessing the association of dog characteristics in dog-exposed children and asthma diagnosis after age 1 in 61,653 children born July 2005-2010.**

|  | **IR per 1000  p-years** | **HR (95% CI)** | **HR**^a^ **(95 % CI)** |
| --- | --- | --- | --- |
| **Dog sex** |  |  |  |
| male | 17 (16-18) | 1.00 | 1.00 |
| female | 17 (16-18) | 0.97 (0.89, 1.06) | 0.98 (0.90,1.07) |
| both | 16 (14-18) | 0.91 (0.80, 1.04) | 0.96 (0.81,1.14) |
| **Breed group** |  |  |  |
| Sheepdogs and Cattledogs | 17 (14-19) | 1.12 (0.94, 1.32) | 1.08 (0.91,1.28) |
| Pinscher and Schnauzer - Molossoid | 18 (16-20) | 1.16 (1.01, 1.35) | 1.12 (0.97,1.30) |
| Terriers | 16 (13-18) | 0.98 (0.82, 1.18) | 0.93 (0.78,1.12) |
| Dachshunds | 14 (10-20) | 0.95 (0.67, 1.37) | 0.96 (0.67,1.37) |
| Spitz and primitive types | 16 (13-18) | 1.04 (0.85, 1.27) | 1.02 (0.83,1.24) |
| Scent hounds and related breeds | 12 (9-15) | 0.78 (0.61, 1.00) | 0.79 (0.62,1.02) |
| Pointing Dogs | 14 (11-18) | 0.95 (0.73, 1.25) | 0.98 (0.75,1.28) |
| Retrievers - Flushing Dogs - Water Dogs | 15 (13-16) | 1.00 | 1.00 |
| Companion and Toy Dogs | 20 (18-23) | **1.27 (1.07, 1.52)** | 1.16 (0.97,1.38) |
| Sighthounds | 18 (12-26) | 1.14 (0.77, 1.69) | 1.11 (0.75,1.65) |
| Cross-breed | 19 (18-21) | **1.17 (1.02, 1.34)** | 1.07 (0.93,1.23) |
| More than one group | 17 (15-19) | 1.04 (0.89, 1.21) | 1.04 (0.86,1.26) |
| **Hypoallergenic breeds – web definition** |  |  |  |
| No | 16 (15-17) | 1.00 | 1.00 |
| Yes | 18 (16-21) | 1.11 (0.97, 1.28) | 1.05 (0.92,1.21) |
| Both | 17 (15-20) | 0.98 (0.84, 1.16) | 0.99 (0.82,1.19) |
| **Hypoallergenic – AKC definition** |  |  |  |
| No | 16 (15-17) | 1.00 | 1.00 |
| Yes | 20 (16-23) | **1.21 (1.01, 1.45)** | 1.11 (0.93,1.33) |
| Both | 17 (14-20) | 0.97 (0.82, 1.16) | 0.96 (0.79,1.17) |
| **Number of dogs** |  |  |  |
| 1 | 17 (16-18) | 1.00 | 1.00 |
| >1 | 16 (15-18) | 0.96 (0.87, 1.05) | 0.97 (0.86,1.09) |
| **Dog size** |  |  |  |
| Small (<40 cm) | 17 (15-18) | 1.03 (0.93, 1.16) | 1.00 (0.89,1.12) |
| Medium (40-60 cm) | 16 (15-17) | 1.00 | 1 |
| Large (>60 cm) | 16 (14-17) | 1.01 (0.89, 1.15) | 1.00 (0.88,1.13) |
| >one size | 17 (15-19) | 1.03 (0.90, 1.18) | 1.07 (0.90,1.28) |

^a^Adjusted for maternal age (<25, 25-29,30-34,≥35), Parents' birth country (Nordic/non-nordic), parental education (max) (Middle school, High school, College(<3yrs), College graduates or higher), parental asthma (yes/no), parental allergy (yes/no), population density (per km^2^), dog sex and number of dogs in the family, when applicable

IR, incidence rate per 1000 person-years, HR, hazard ratio, CI, confidence interval

**eTable 4. Logistic regression models assessing the association of dog characteristics in 10,954 first-born dog-exposed children and asthma diagnosis at age six.**

|  | **Asthma n (%)** | **OR (95% CI)** | **OR^a^ (95% CI)** |
| --- | --- | --- | --- |
| **Dog sex** |  |  |  |
| male | 335 (5.8) | 1.00 | 1.00 |
| female | 231 (5.5) | 0.95 (0.80, 1.13) | 0.95 (0.80,1.13) |
| both | 50 (5.3) | 0.91 (0.67, 1.24) | 1.00 (0.65,1.52) |
| **Breed group** |  |  |  |
| Sheepdogs and Cattledogs | 70 (4.9) | 0.76 (0.56, 1.02) | **0.71 (0.53,0.96)** |
| Pinscher and Schnauzer - Molossoid | 123 (6.7) | 1.04 (0.81, 1.34) | 1.01 (0.79,1.30) |
| Terriers | 30 (4.0) | **0.62 (0.41, 0.92)** | **0.58 (0.39,0.87)** |
| Dachshunds | 8 (2.9) | **0.44 (0.21, 0.90)** | **0.43 (0.21,0.90)** |
| Spitz and primitive types | 41 (5.6) | 0.88 (0.61, 1.25) | 0.85 (0.59,1.22) |
| Scent hounds and related breeds | 40 (5.4) | 0.85 (0.59, 1.21) | 0.82 (0.57,1.18) |
| Pointing Dogs | 17 (3.6) | **0.56 (0.33, 0.93)** | **0.55 (0.33,0.93)** |
| Retrievers - Flushing Dogs - Water Dogs | 145 (6.3) | 1.00 | 1 |
| Companion and Toy Dogs | 49 (7.5) | 1.19 (0.85, 1.67) | 1.07 (0.76,1.51) |
| Sighthounds | 6 (4.9) | 0.76 (0.33, 1.75) | 0.77 (0.33,1.81) |
| Cross-breed | 45 (5.8) | 0.90 (0.64, 1.28) | 0.87 (0.61,1.25) |
| More than one group | 42 (5.1) | 0.79 (0.55, 1.12) | 0.79 (0.52,1.21) |
| **Hypoallergenic - web definition** |  |  |  |
| No | 504 (5.6) | 1.00 | 1.00 |
| Yes | 56 (6.2) | 1.12 (0.84, 1.49) | 1.05 (0.78,1.40) |
| Both | 11 (4.7) | 0.84 (0.45, 1.55) | 0.87 (0.46,1.64) |
| **Hypoallergenic – AKC definition** |  |  |  |
| No | 530 (5.6) | 1.00 | 1.00 |
| Yes | 34 (6.9) | 1.26 (0.88, 1.80) | 1.10 (0.77,1.59) |
| Both | 7 (3.8) | 0.67 (0.31, 1.42) | 0.69 (0.32,1.50) |
| **Number of dogs** |  |  |  |
| 1 | 499 (5.8) | 1.00 | 1.00 |
| >1 | 117 (5.2) | 0.89 (0.72, 1.09) | 0.88 (0.68,1.15) |
| **Dog size** |  |  |  |
| Small (<40 cm) | 127 (5.7) | 0.97 (0.79, 1.21) | 0.96 (0.77,1.19) |
| Medium (40-60 cm) | 295 (5.9) | 1.00 | 1 |
| Large (>60 cm) | 122 (5.5) | 0.94 (0.76, 1.17) | 0.94 (0.75,1.17) |
| >one size | 27 (3.9) | **0.66 (0.44, 0.99)** | 0.67 (0.43,1.06) |

^a^Adjusted for maternal age (<25, 25-29,30-34,≥35), parents' birth country (Nordic/non-nordic), parental education (max) (Middle school, High school, College(<3yrs), College graduates or higher), parental asthma (yes/no), parental allergy (yes/no), population density (per km^2^), dog sex and number of dogs in the family, when applicable
